# Spectrum of imaging findings in Takayasu arteritis—A case report^☆^^☆☆^

**DOI:** 10.1016/j.radcr.2022.06.092

**Published:** 2022-07-27

**Authors:** Kritisha Rajlawot, Sujan Thapa, Asim Sitaula, Nirmal Prasad Neupane

**Affiliations:** Department of Radiodiagnosis and Imaging, Shahid Gangalal National Heart Centre, Bansbari, Kathmandu, Nepal

**Keywords:** Takayasu arteritis, Ultrasonography, Doppler study, CT angiography

## Abstract

Takayasu arteritis (TA) is an uncommon chronic granulomatous inflammatory disease often affecting the aorta and its branches. Early diagnosis is quite challenging due to nonspecific symptoms and unfamiliarity with the disease. We hereby present a case of a young female patient diagnosed with Type V Takayasu arteritis using several radiological imaging modalities such as color and spectral Doppler study and computed tomography angiography. A timely diagnosis of Takayasu arteritis however may improve the outcome such as irreversible target organ damage and poor prognosis with a decreased rate of complications.

## Introduction

Takayasu arteritis (TA) is an uncommon chronic granulomatous inflammatory disease that majorly affects female patients often between 20 and 40 years of age [1]. The aorta and its branches are frequently affected, not always but TA may also involve proximal portions of pulmonary, coronary, and renal arteries. It disrupts the arterial endothelium, eventually resulting in stenosis, endoluminal thrombosis, and aneurysmal dilatation [2]. Being a rare variety of vasculitis, early diagnosis is quite challenging due to nonspecific symptoms and unfamiliarity with the disease. Hence, it is a must to avoid a late diagnosis and delayed treatment so as to null the morbidity and mortality caused by the condition. We hereby present a case of a young female patient diagnosed with Type V Takayasu arteritis showing multiple vessel involvement.

### Clinical history

A 21-year-old female patient presented to our Out-Patient Department with complaints of headache, uncontrolled hypertension, and weakness in her upper limbs for a year. On general examination, her pulse on the left upper limb was not detected with immeasurable blood pressure. The patient was therefore sent for arterial Doppler of both upper limbs for evaluation. No significant family history was given. No other symptoms such as fever, nausea, vomiting, or visual problems were given.

### Imaging findings

**Fig. 1:**
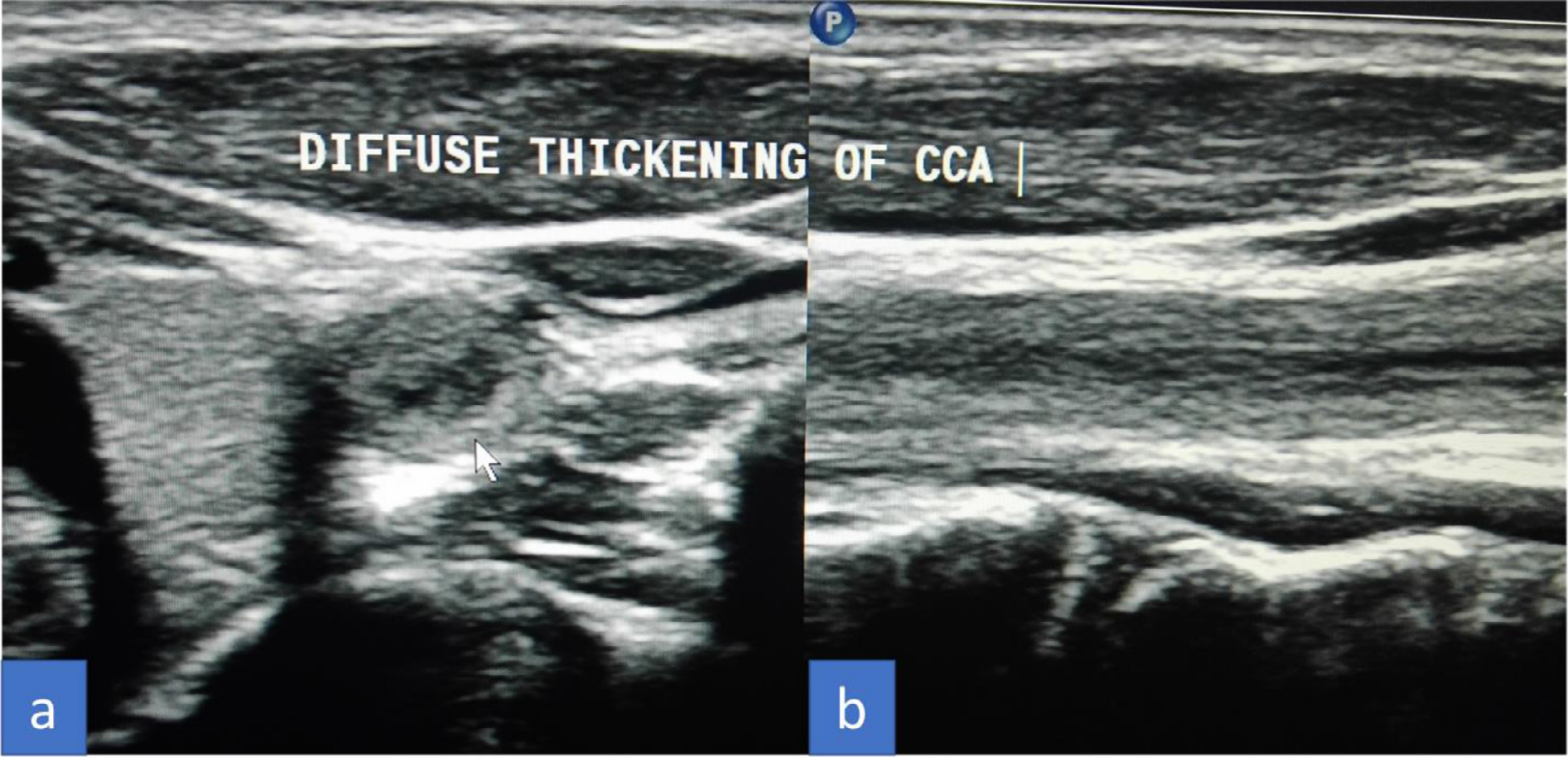
Ultrasonography of common carotid artery (CCA) transverse image (A), longitudinal image (B) showing diffuse circumferential thickening of CCA.

**Fig. 2:**
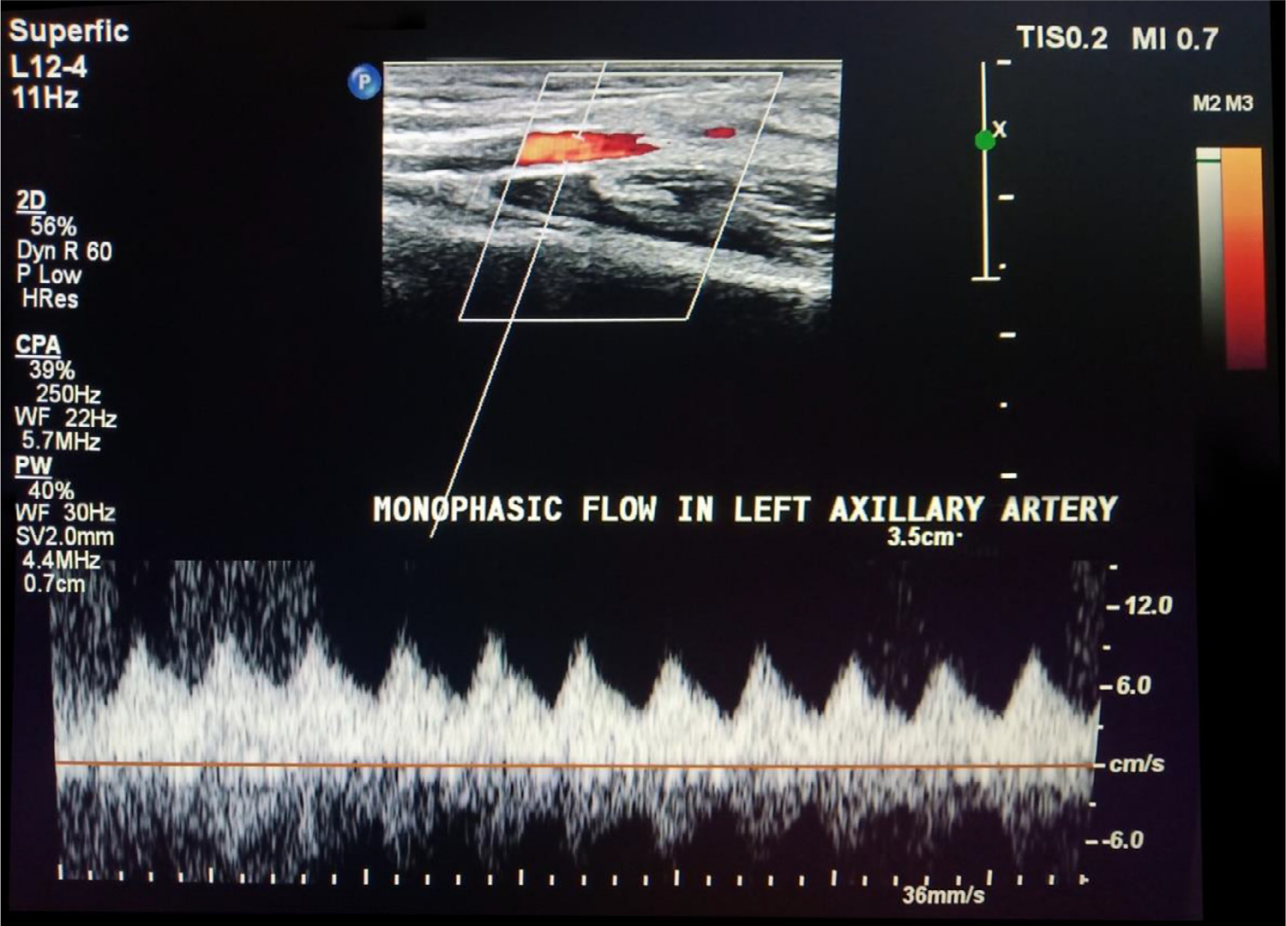
Spectral Doppler study of the left axillary artery.

**Fig. 3:**
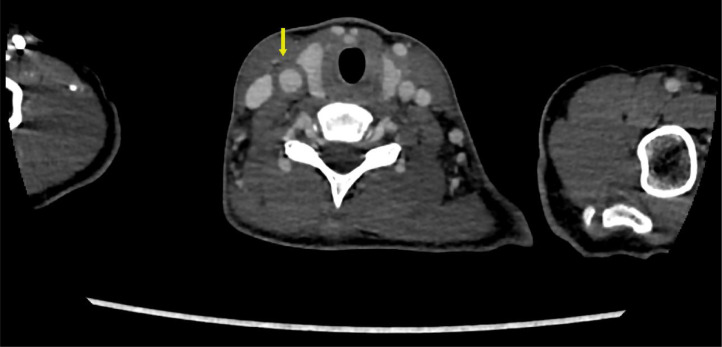
CTA axial view showing a diffusely thickened wall of right CCA (arrow).

**Fig. 4:**
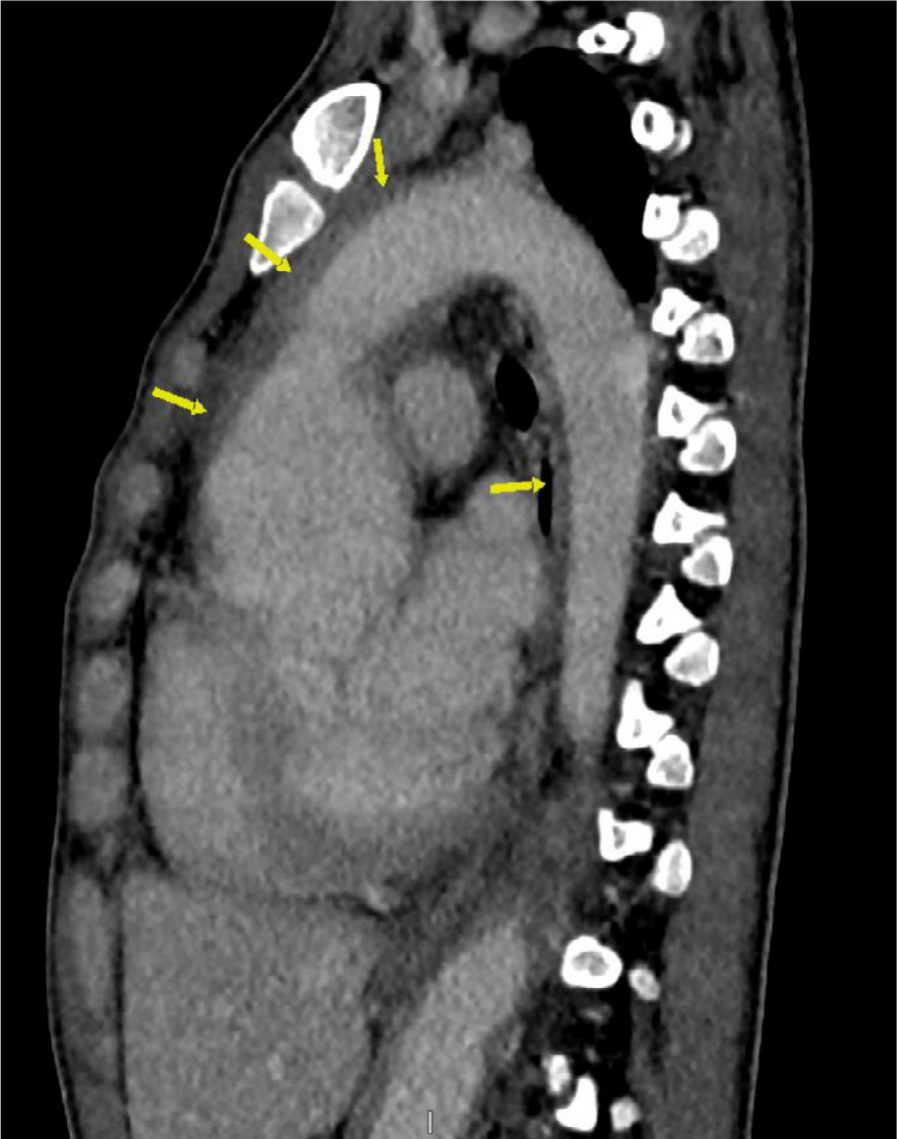
CTA sagittal view showing a diffusely thickened wall of ascending aorta and major arch vessels along with distal descending thoracic aorta and abdominal aorta (arrow).

**Fig. 5:**
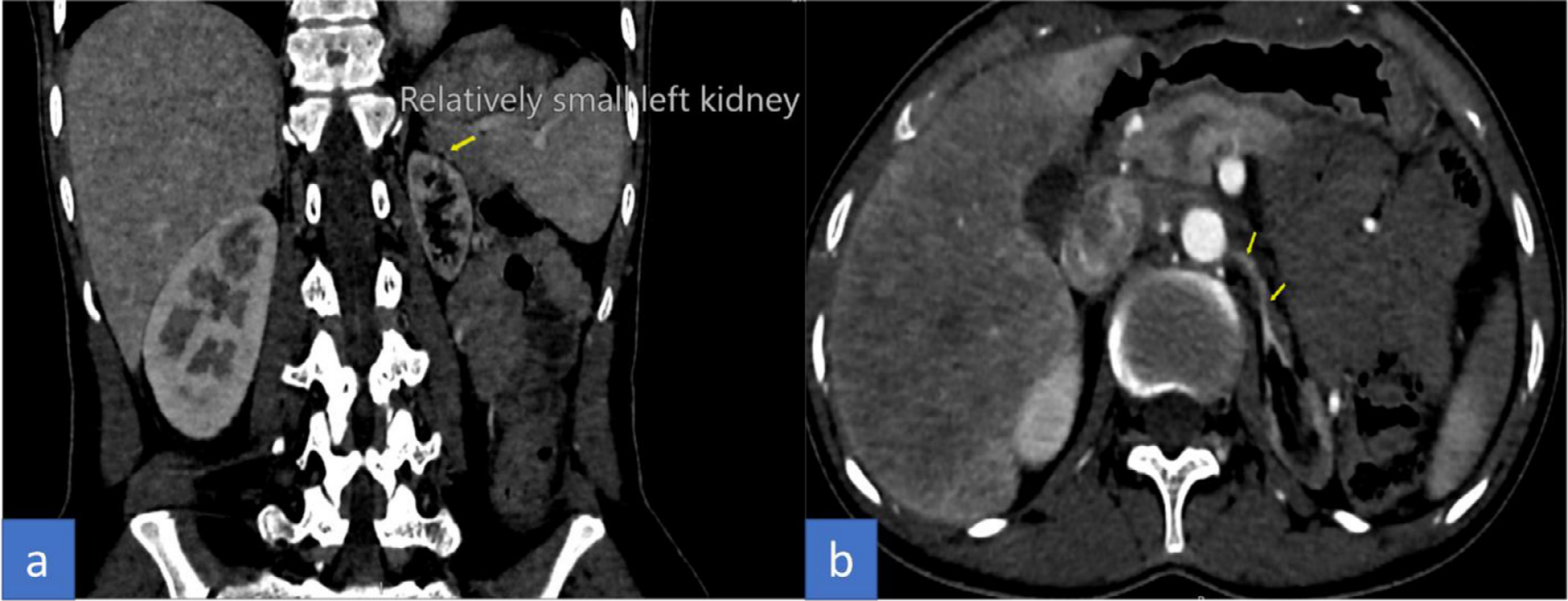
CTA coronal view (A), axial view (B) showing small left kidney with marked thickening of the left renal artery, narrowing of its lumen, and poor opacification of the contrast (arrow).

**Fig. 6:**
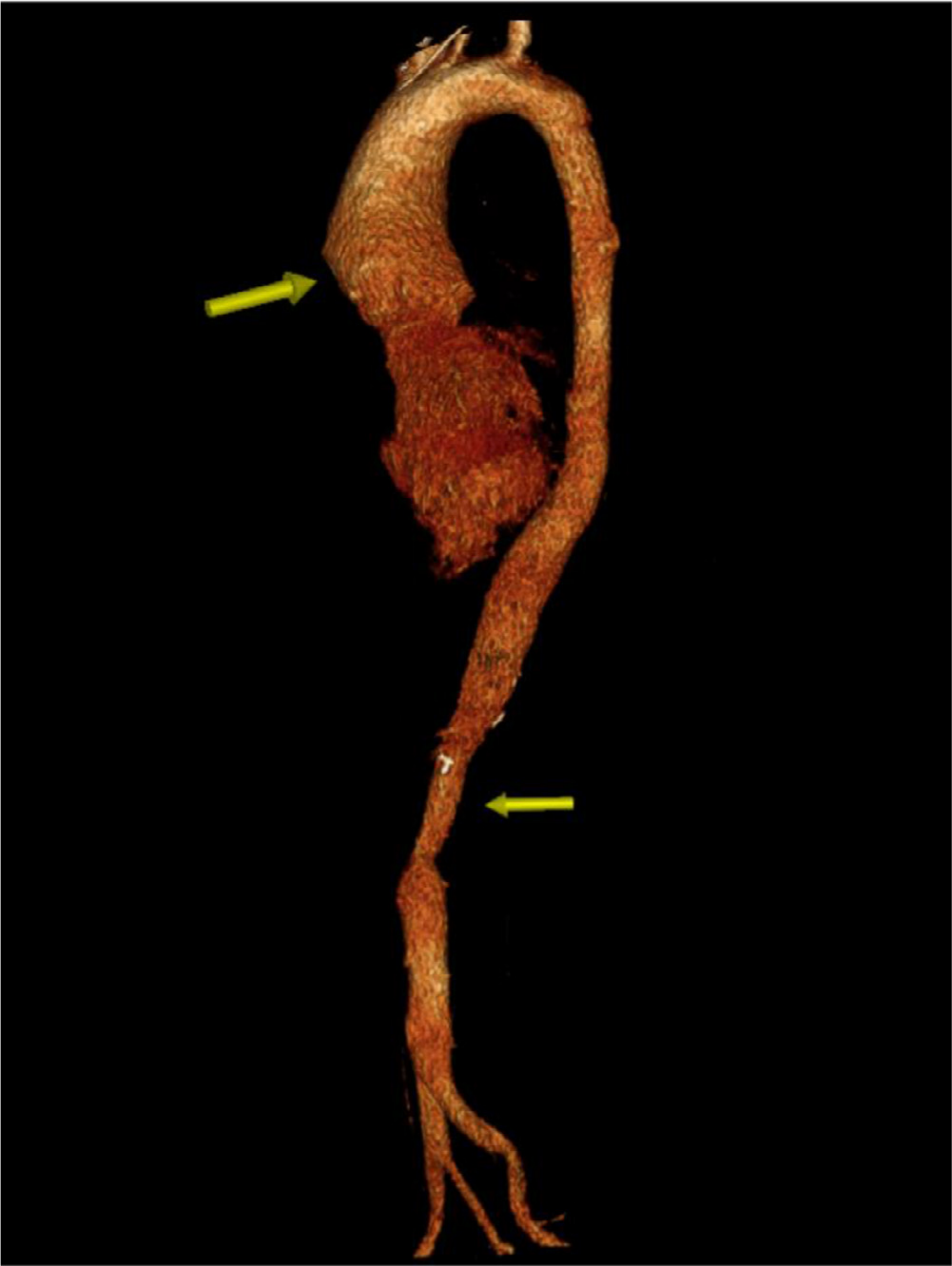
Volume rendered image showing aneurysmal dilatation of ascending aorta with focal narrowing of the abdominal aorta (arrows).

The patient initially underwent arterial Doppler of upper limbs for weakness. In the Doppler study, diffuse circumferential thickening of both common carotids was noted giving off a significant “macaroni sign” (Figs. 1A and B). The spectral Doppler study showed a monophasic waveform with markedly reduced velocities in the left axillary, brachial, radial, and ulnar arteries (Fig. 2). The patient was advised for a multidetector CT aortogram for further evaluation. CT aortogram showed diffuse circumferential wall thickening without calcification in bilateral carotid arteries with marked narrowing of the subclavian vessels distally (Fig. 3). There was a diffusely thickened wall of ascending aorta and major arch vessels along with distal descending thoracic aorta and abdominal aorta (Fig. 4). Fusiform dilatation of the ascending aorta was noted that was extending and involved the aortic root (Fig. 6). There was a marked thickening of the left renal artery with narrowing of its lumen and resultant smaller left kidney with poor opacification of the contrast (Figs. 5A and B). Based on the vessels involved in imaging findings, Type V Takayasu arteritis was reported.

**Table 1:** Angiographic classification of Takayasu arteritis [5,6].

Type	Vessel involvement
I	Branches from the aortic arch
IIa	Ascending aorta, aortic arch and its branches
IIb	Ascending aorta, aortic arch and its branches, thoracic descending aorta
III	Thoracic descending aorta, abdominal aorta, and/or renal arteries
IV	Abdominal aorta and/or renal arteries
V	Combined features of types IIb and IV

## Discussion

Takayasu arteritis also termed as “pulseless disease” or “idiopathic medial aortopathy” is a nonspecific inflammation of arteries causing concentric wall thickening, fibrosis, vessel stenosis, occlusion, and formation of aneurysm or thrombus [1,3]. The symptoms of Takayasu arteritis vary and can be classified into 2 phases: an initial phase including nonspecific symptoms like fever, malaise, and headache, and a late stenotic phase with symptoms like hypertension, claudication, pulselessness, bruit, and angina. Hypertension is a common manifestation present in 80 % of the cases as a result of renal artery stenosis [4]. So was the presentation in our case with markedly narrowed left renal artery which got evaluated well through CT angiographic studies. Similarly, the weakness in the upper limbs with absent pulses was well explained by the CT angiographic findings of diffuse circumferential wall thickening in bilateral carotid arteries along with the narrowing of distal subclavian arteries. Depending on the location of the involved vessels, Takayasu arteritis has been divided into 5 types [5,6] (Table 1).

The broad and variable involvement of the aorta and its branches in the same patient, as seen in our patient, is exceptional among the 5 kinds of Takayasu arteritis. Moreover, our current case has enlightened the relevance of several radiological imaging modalities in demonstrating a spectrum of vascular alterations that may be encountered in a single patient suspected of Takayasu arteritis.

## Conclusion

Takayasu arteritis is an uncommon vascular disease that still challenges the physicians to diagnose the disease only depending on the clinical features. However, though rare, with a timely diagnosis of Takayasu arteritis through radiological modalities such as CT angiography, the outcome such as irreversible target organ damage and poor prognosis may be improved with a decreased rate of complications.
